# Animal Individual Factors Dictate Learning Strategy: An Integrated Analysis of Cattle Adaptation to a Virtual Fencing System

**DOI:** 10.3390/ani16172732

**Published:** 2026-09-02

**Authors:** Severino Pinto, Clemens Bodt, Julia Toups, Dina Hamidi, Gundula Hoffmann

**Affiliations:** 1 Department of Sensors and Modelling, Leibniz Institute for Agricultural Engineering and Bioeconomy—ATB, Max-Eyth-Allee 100, 14469 Potsdam, Germany; 2 Finck Stiftung gGmbH, Schlossstr. 19, 15518 Briesen (Mark), Germany; clemens@gutundboesel.org (C.B.); julia@gutundboesel.org (J.T.); 3 Department of Crop Sciences, Grassland Science, University of Göttingen, Von-Siebold-Str. 8, 37075 Göttingen, Germany; dina.hamidi@uni-potsdam.de; 4 Institute for Biochemistry and Biology, University of Potsdam, Maulbeerallee 1b, 14469 Potsdam, Germany

**Keywords:** animal learning, cattle behaviour, age effect, individual variation, smart farming

## Abstract

Virtual fencing (VF) is a promising technology for pasture management, but its successful implementation depends on understanding the factors that influence the animal learning. The aim of the present study was to evaluate the significance of individual animal factors that affect the behavioural and performance-based learning process in cattle during a VF training period. We analysed collar-derived performance data from 171 mixed-age Angus and Salers breed cattle over a 12-day training trial. Learning performance was primarily measured by daily success ratio (SR) and confidence ratio (CR). Learning trajectories and the influence of animal factors were analysed using linear mixed-effects models. A significant increase was observed in the SR over the 12-day period (*p* < 0.0001). A significant interaction between training day and age class (*p* < 0.001) revealed distinct learning strategies: younger animals showed rapid initial adaptation but lower consistency, whereas adult animals exhibited a more stable final performance. Our findings suggest that breed may also influence the learning rate to virtual fencing (*p* < 0.001). Individual behaviour accounted for a substantial portion of performance variation (*p* < 0.0001). Recognising these different individual and social learning pathways is essential for developing more effective and improved animal welfare VF training protocols.

## Simple Summary

Virtual fencing is a technology that uses GPS collars to manage cattle on pasture without physical fences. The collar emits an audio cue when the animal approaches the virtual boundary, and if it does not turn back to the inclusion zone, it subsequently delivers a mild electrical pulse. This study aimed to evaluate the influences of individual animal factors on the learning process in beef cattle during a virtual fencing training period. A total of 171 beef cattle with GPS collars were monitored over a 12-day training period and their performance was tracked by calculating a success ratio. The cattle have learned successfully; however, the age and breed were important influencing factors. Young animals showed rapid initial adaptation but lower consistency, whereas adult animals exhibited a more gradual but ultimately higher and more stable final performance. Angus cattle showed a higher success ratio in comparison with Salers. However, this finding might be more related to individual ability in adapting to virtual fencing. Differences between individual animals were also observed during the training. These findings suggest that for a training protocol to be effective, it must consider individual animal factors that influence adaptation to virtual fencing.

## 1. Introduction

Grazing systems are fundamental to land use conservation and animal welfare in cattle production [1]. However, the economic viability of these systems hinges on a direct comparison with the high costs of physical fencing and labour, and the sustainability of these systems can be compromised, representing an ongoing challenge for producers. Virtual fencing (VF) technology serves as a GPS-based enclosure that creates an invisible boundary, which can increase flexibility while potentially reducing labour, improving herd management, and protecting environment-sensitive extensive areas [2,3,4]. In recent decades, this technology has been improved with companies worldwide developing proprietary systems. These devices, which are commercially available and widely used on-farm in some regions of world, aim to replace traditional physical fences by an alternative flexibility device [5,6]. The companies utilise similar devices for diverse clients to set the virtual boundaries, usually solar-powered GPS collars worn by the animals that are able to influence their behaviour by emitting an audio cue and a mild electrical pulse.

Implementation requires a training period based on associative learning, where a neutral audio cue is paired with an aversive electrical pulse if an animal continues toward the boundary [7,8]. The learning trajectory of cattle to a VF involves a transition process. Initially, through avoidance learning, animals rapidly learn to remain within the specified inclusion zone by responding to repeated audio–pulse combinations [9]. This is followed by associative learning, where the animals respond appropriately to the audio cue alone and are able to prevent the electrical pulse [10,11]. The duration of this learning process varies between individuals, though research indicates that cattle typically require between one and six fence interactions and learn to respond appropriately to the virtual boundary within 2 to 4 days [5,12,13].

Recent studies have established standardised metrics to evaluate the learning process of cattle with virtual fencing systems [9,14]. The success ratio (SR) for assessing the learning capability of cattle is calculated as the proportion of audio cues without a following electrical pulse relative to all collar cues. The studies indicate that cattle respond to the VF by returning to the virtual enclosure after hearing the auditory cue without triggering the electrical cue [4]. To address the limitations of the SR, the confidence ratio (CR) has been proposed to reflect an animal’s ability and willingness to successfully interact with virtual boundaries. This metric assesses proficiency against a benchmark of 20 successful interactions. It is calculated by multiplying the SR by the number of successful audio cues (capped at a maximum of 20) and then dividing this benchmark by 20. This approach rewards animals that not only respond correctly but do so frequently. Previous studies have demonstrated that the learning trajectories for the SR and CR follow different timelines [4,9]. While the SR, which measures initial avoidance learning, often increases significantly within the first two days, the CR typically does not increase until later training phases (e.g., between day 6 and 7). This was attributed to increased familiarity with the fence location, indicating the desired understanding of the VF by the cattle and a reduction in fear of interaction [4,9].

The behaviour of cattle during the training period has revealed minor and inconsistent differences in previous grazing trials [13,15], especially in grazing and rumination time [16]. Several studies have applied behavioural scoring during the training period of virtual fencing, which demonstrates a range of responses from low to high intensity [9,16], in some cases using camera support for animal evaluation [16]. Most of these studies, however, evaluated a uniform group of animals or small herds to maintain observational control, making it challenging to quantify the impact of individual animal variation in such groups.

Adaptation to virtual fencing has been reported with a large variation between individuals. The learning rate is not uniform, with some animals interacting with the fence more frequently than others [5,9,12]. These effects can be related to several individual animal factors, including behavioural personality, age, and breed. Animals with a more proactive or bold behavioural style may be more likely to approach and test the virtual boundary, whereas more reactive or shy animals may avoid it. Such differences could affect both the number of warnings and electrical stimuli received and the apparent rate of learning [13,17]. Controversial findings have been observed regarding age: Verdon and Rawnsley [7] found that 22-month-old heifers learned faster than younger calves, while Confessore et al. [18] found that young (first lactation) and old (>four lactations) cows adapted equally fast to VF, although younger cows were significantly more active. Furthermore, various beef and dairy breeds have demonstrated the ability to learn the VF system, including Santa Gertrudis [3], Rarámuri Criollo [19], Limousin [20], Fleckvieh [15], Holstein–Friesian [13], and the most common Angus [5,17]. There are no studies that have used the Salers breed or directly compared two beef breeds during the same training period. While some studies have used multi-breed herds, they have typically been analysed as a single experimental unit rather than comparing breeds against each other [21,22].

Most recent studies have been conducted with small groups (from four to twelve animals) under controlled experimental settings [15,17,23]. A thorough understanding of cattle’s social and herd behaviour is essential for determining the optimal management for VF, as social facilitation plays a critical role [24]. Because cattle are herd animals that exhibit strong social learning, a substantial proportion of the herd may learn to recognise and respond to virtual boundaries by observing others. Therefore, evaluating a large and heterogeneous herd may reveal more details of VF adaptation. To the best of our knowledge, there is a lack of research evaluating the implementation of VF in large, commercially relevant herd sizes.

Therefore, the aim of the present study was to evaluate the behavioural responses using a camera drone and the influence of individual animal factors on successful learning during the adaptation period of cattle to a VF system under grazing conditions. Specifically, we approached the influence of intrinsic animal factors on the learning trajectory in a large herd. We hypothesised that: (H1) the frequency and intensity of adverse behavioural reactions recorded by the camera drone would significantly decrease over the initial training period; (H2) performance metrics such as the SR and CR would increase over the training period, indicating successful learning; (H3) the rate of learning would be influenced by individual animal factors, e.g., age and breed, explaining a significant portion of the variation in the learning trajectories; and (H4) a higher intensity of initial aversive stimuli (i.e., more electrical pulses during the adaptation phase) would be correlated with a faster subsequent learning rate.

## 2. Materials and Methods

### 2.1. Animals and Management

The present study was conducted in April 2024 on a certified ecological farm, located in eastern Brandenburg, Germany (52.2° N, 14.1° E). The trial herd consisted of 171 beef cattle from a larger group of 187 animals including calves, heifers, ox, and mother cows. The selected cohort included purebred Angus (*n* = 125) and Salers (*n* = 46), with a median age population of 22.89 months (Interquartile Rage—IQR: 11–60 months). Prior to the trial, animals weighing over 300 kg were selected and underwent a clinical examination, including Body Condition Scoring (BCS). For analysis, animals were categorised into three age classes based on developmental stage: young (<13 months, *n* = 60), middle age (13–24 months, *n* = 38), and adult (>24 months, *n* = 73). All animals were managed under a pasture-based system and had prior experience with conventional electric fencing but were naive to virtual fencing (VF) technology.

### 2.2. Virtual Fencing System and Habituation Period

Each animal was fitted with a commercial eShepherd^®^ neckband collar (Gallagher Group Limited, Hamilton, New Zealand), weighing approximately 3 kg. The system comprises a GPS-enabled collar, a solar-rechargeable battery, a mobile network connection for data record and transmission every minute to a cloud-based server, and a user interface (eShepherd App of iOS and Android, version 2.28.0.). The VF system functions by delivering an auditory cue for 2.5 s (an ascending tone at 785 Hz ± 15 Hz, 58 dB at 1 m) when the animals approach the boundaries according to the GPS location signalling to the animal to stop walking in that direction. If the animal does not retreat, the collar delivers a short, localised electrical stimulus of 800 V in for less than 1 s [5]. Additional technical parameters of the stimulus are considered proprietary information by the manufacturer. The animal can stop the audio cue and avoid the pulse by turning its head away from the boundary. If the animal ignores the audio cue and the following electric pulse, after three successive audio cue–pulse pairs, the system considers that animal as “escaped”, deactivates stimuli, notifies the user via app, and the animal’s location can be monitored automatically by GPS. Once the animal re-enters the inclusion zone, the system resumes normal functionality. Four weeks before the trial started, the selected animals were equipped with the collars (in a deactivated state) and habituated to the 2-ha trial paddock, with access to hay and water ad libitum, to acclimatise to the collar’s weight and management routine.

### 2.3. GPS Accuracy Assessment

To evaluate the GPS accuracy of the VF system, three eShepherd^®^ collars were assessed by comparing recorded positions with known reference points and quantifying the spatial deviations. A 50-metre-long route was defined adjacent to the test site, the coordinates of which were measured using a high-precision GPS device. The collars were carried by a test person along the surveyed route for 1 h and subsequently placed on the route for an additional 30 min, yielding *n* = 270 GPS points. Deviations from the predefined track were calculated and interpreted as an estimate of average GPS inaccuracy. These metrics were used to assess system performance and to provide context for, and partly explain, the observed behavioural responses of the animals.

During movement along a known reference track, the mean spatial deviation was (mean ± SD) 1.18 ± 0.47 m, whereas during the stationary test, the deviation was (mean ± SD) 0.99 ± 0.29 m. These results indicate a high level of spatial precision for the VF system under the conditions of our study.

### 2.4. Experimental Design and Training Protocol

The 12-day training trial was conducted in the paddock enclosed by perimeter physical wire-based fence with an electrified wire. As part of the training, a portable single-electrified poly wire was installed beyond the virtual boundary in a distance according to the training day. This backup fence was intended to contain any escaped animal that might breach the virtual fence. The VF was active during the daylight hours and deactivated at night with supervision of a trained person.

**Figure 1:**
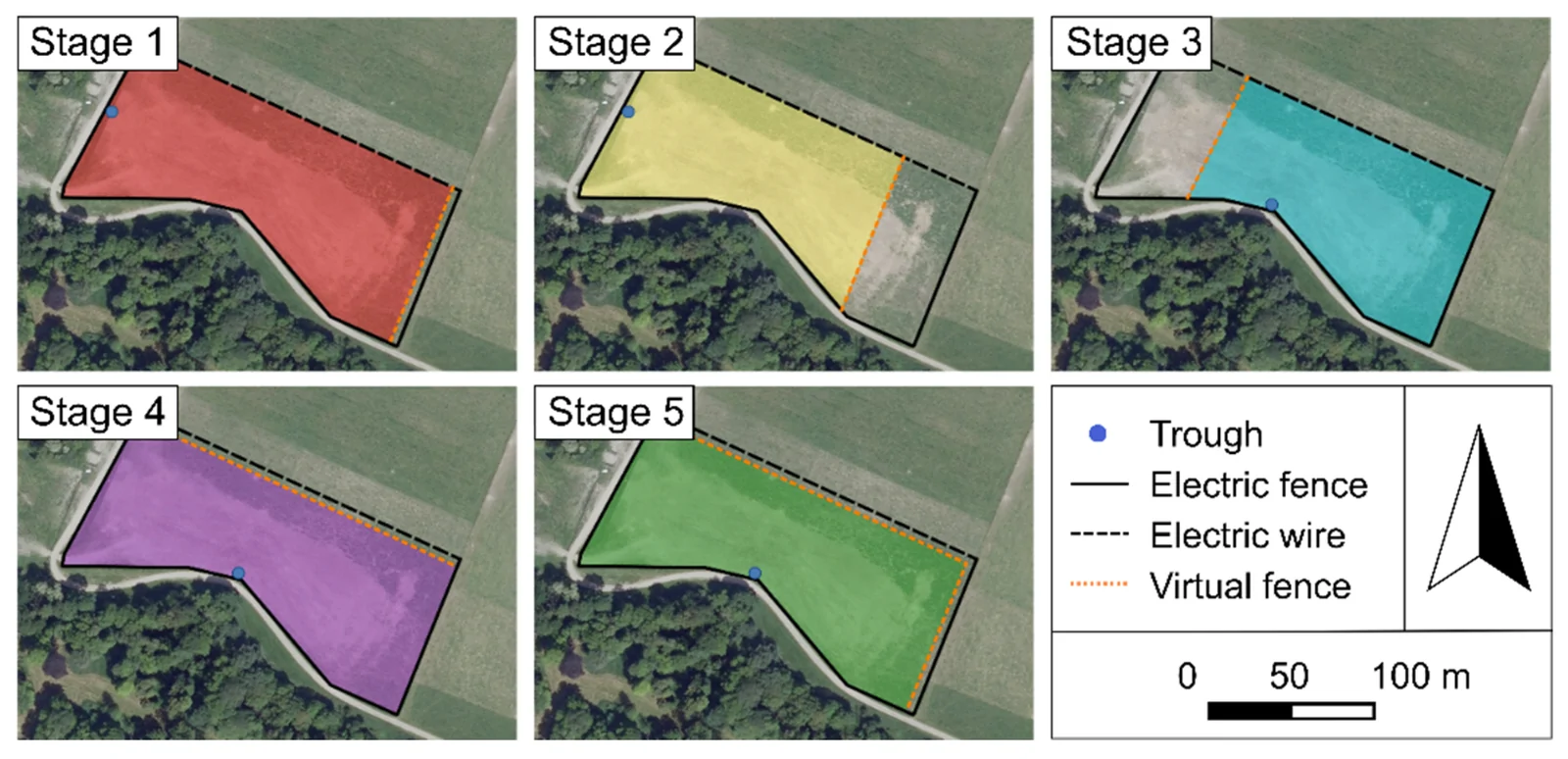
The paddock set up enclosed with physical electric wire-based fence (black line) and electric poly-wire fence (black striped line) used in the trial during the training period. The virtual fence line is indicated with an orange dotted line within test stages. Stage 1 (red, Day 1): VF 5 m from the physical fence; Stage 2 (yellow, Days 2–3): VF 50 m from the physical fence; Stage 3 (turquoise, Days 4–5): VF 50 m from the physical fence; Stage 4 (purple, Day 6): VF 5 m from electric poly wire on the long side of the paddock; and Stage 5 (green, Days 7–12): VF as a combination of Stages 1 and 4.

The trial was structured into five progressive stages of training designed to teach generalisation and test-learning robustness (Figure 1): Stage 1 (Day 1) provided the initial association, with the VF line placed 5 m from the physical electric wire fence. To teach generalisation of distance, Stage 2 (Days 2–3) was with the VF line moved to 50 m from the electric fence, requiring animals to rely solely on collar cues. To teach generalisation of location, Stage 3 (Days 4–5) was with the VF line placed 50 m from the electric fence on the opposite side of the paddock, demonstrating that the boundary was dynamic and not fixed to one area. Stage 4 (Day 6) challenged the animals, with the VF line placed on the long side of paddock, 5 m from the portable single electrified poly wire. Finally, Stage 5 (Days 7–12) tested the retention of these generalised rules in a more complex environment, with the VF line configured with a combination of Stage 1 and 4, designed to progressively challenge and reinforce the desired avoidance behaviour. For analytical purposes, these stages were conceptually grouped into three learning phases: Adaptation phase (Stages 1–2), which focused on the initial association between the audio cue and the electrical pulse, Consolidation phase (Stages 3–4), aiming to reinforce and solidify the learned avoidance response, and Validation phase (Stage 5), which assessed the long-term retention and stability of the learned behaviour.

### 2.5. Data Collection and Processing

#### 2.5.1. Behavioural Data from Drone Footage

During the initial training stages (Days 1–5), animal behavioural reactions to VF stimuli were recorded using the camera drone DJI Mavic III (SZ DJI Technology Co., Ltd., Shenzhen, China) at a resolution of 4K at 30 fps. Multiple video samples (12–16 records per day) of approximately 20 min were captured from a height of 30 m, with filming timed opportunistically throughout the day to observe the virtual fence area and the animals that reach the boundary and get the audio cue and electrical pulse. As the herd comprised a lot of animals, a precise visual identification for the behaviour scoring of each animal was not feasible from the footage. These observations were categorised by group characteristics visible from the footage: age class (Young vs. Adult). The videos were analysed later, and the reactions were classified on an ordinal scale adapted from Hamidi et al. [9]. The reaction scores of the animals in contact with VF are demonstrated in Table 1.

#### 2.5.2. Collar-Derived Performance Data

Collar data, including the timestamp and location of every audio cue, and electrical pulse were provided by eShepherd^®^. As part of the preprocessing, raw data were screened for invalid measurements and artefacts were removed.

Key performance metrics were calculated for each animal; the success ratio (SR) according to Eftang et al. [14] and the confidence ratio (CR) according to Hamidi et al. [9]. Success (S) assesses an instance where an animal responded to an audio cue without receiving a subsequent electrical pulse (S = Number of audio cues − Number of electric stimuli) [14]. This value was then capped at a maximum of 20 acoustic signals without a subsequent electric pulse during the adaptation period of VF (S′ = 0 ≤ S ≤ 20) [9]. The daily SR as the primary metric of learning was then calculated for each animal using the success value divided by the sum of acoustic signals per day. The values could range between 0 and 1, where 1 indicates perfect avoidance using only the audio cue.

$$Success\;ratio=\frac{Number\;of\;audio\;cues-Number\;of\;electric\;stimuli}{Number\;of\;audio\;cues}$$

Furthermore, the daily CR was calculated using the formula:

$$Confidence\;ratio=\frac{Success\;ratio\times S^{\prime }}{20}$$

This results in a CR value of 1.0 when an animal achieves a perfect SR and has 20 or more successful responses, indicating a high level of confidence and learning according to this criterion.

### 2.6. Statistical Analysis

All statistical analyses were conducted using JMP^®^ Pro 19.1.4 (SAS Institute Inc., Cary, NC, USA), with significance declared at α < 0.05. Observational data of behavioural responses to the virtual fence were analysed separately from the collar data. Herd-level habituation over the first five days was assessed using an ordinal logistic regression model with reaction score as the response and training day as the effect. A descriptive analysis was carried out to analyse learning trajectories, a linear mixed-model effect was used, with daily AC, EP, SR, and CR as the response variables. Training day, age class, and breed were included as fixed effects, along with their two-way interactions. An animal random effect was included to account for the non-independence of repeated measures. Post hoc pairwise comparisons between least-squares means were conducted using Tukey’s HSD adjustment to control for multiple comparisons. The normality of the residuals was checked by visual inspection of quantile–quantile plots. Two metrics were calculated to assess individual learning. The “learning rate” was defined as the slope of the simple linear regression of daily SR vs. training day (Days 3–12) for each animal. The individual “cost to learn” was defined as the cumulative number of electrical pulses an animal received up to the first day it achieved an SR of ≥80%. Differences in the mean “cost to learn” between age class and breed groups were assessed using standard least-square means model (ANOVA). To test the hypothesis that initial training intensity influences learning speed, a simple linear regression was performed to examine the relationship between the “learning rate” (calculated for Days 3–12) and the number of initial electrical pulses (Days 1–2).

## 3. Results

### 3.1. Behavioural Reactions During the Adaptation Phase

Observational data collected by drone during the first five days of training showed a tendency of a habituation effect. The training day had a significant effect on the distribution of reaction scores over the five-day measurement period (Ordinal Logistic Regression; χ^2^(5) = 13.51, *p* < 0.001). A detailed examination of the parameter estimates showed that the most substantial change occurred between Day 3 and Day 4 with a highly significant decrease in higher-intensity reactions scores (Estimate = −1.56, *p* = 0.0009). This indicates a key learning event after the third day of training. Conversely, a significant increase in adverse reactions was observed from Day 4 to Day 5 (Estimate = 1.38, *p* = 0.002), corresponding to a new challenge in the training protocol, interacting more with the VF.

**Figure 2:**
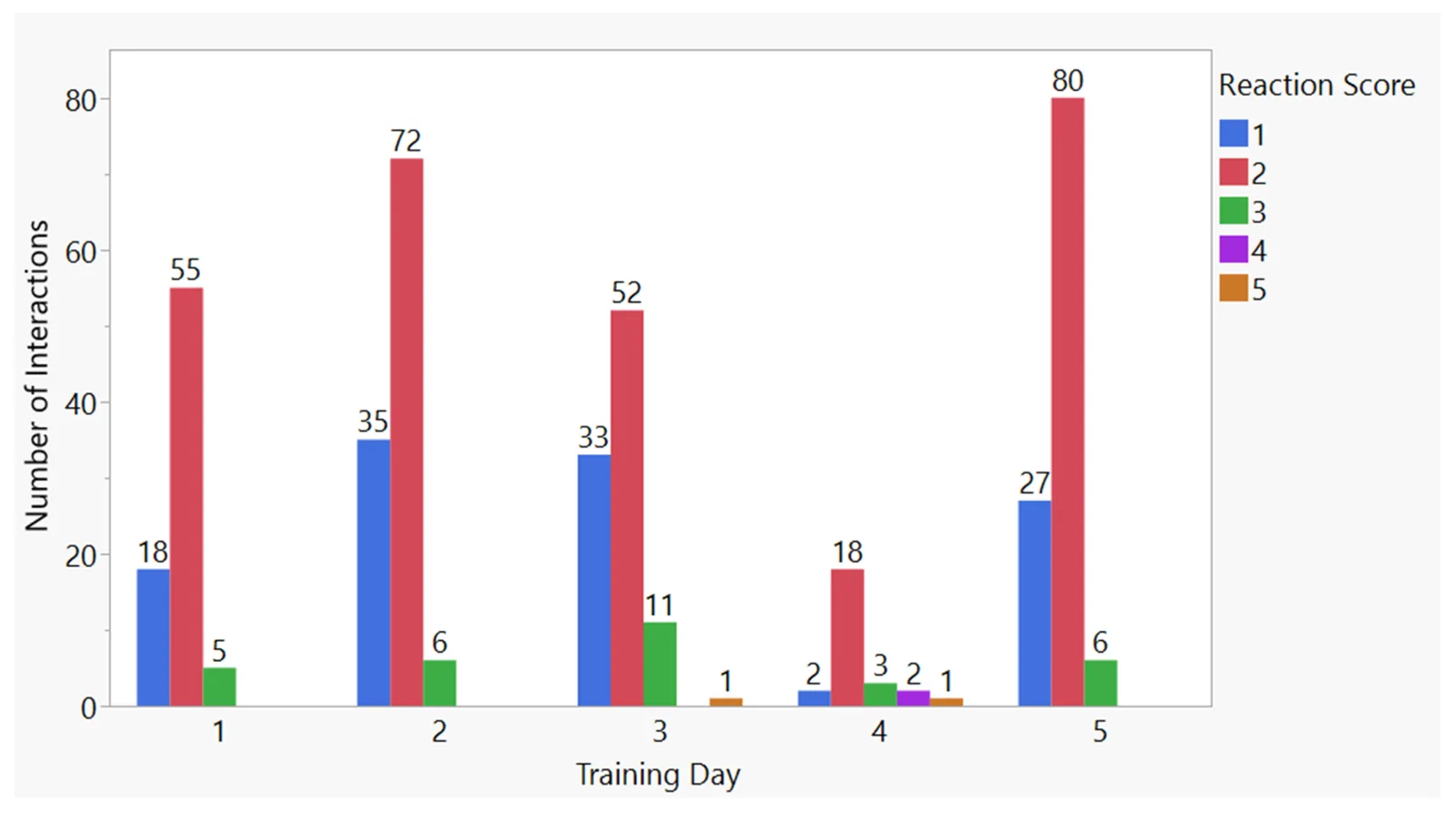
Number of observations from drone footage of cattle behaviour score with contact of virtual fencing during the first five training days.

Figure 2 demonstrates the number of observations from the drone footage of cattle behaviour in each reaction score with contact of virtual fencing during the first five days of training. No significant effect of age class and sex was observed in the reaction scores of the animals during the training (*p* > 0.49).

### 3.2. General Training Effectiveness and Individual Variation

During the 12-day trial, a total of 3817 audio cues and 1544 electrical pulses were delivered from the collars across the herd, demonstrating widespread interaction with VF. Almost all animals (97.66%) had contact with the system, with only four individuals receiving neither an audio cue nor an electrical pulse during the entire study period. The intensity of interaction with the fence varied substantially between animals (*p* < 0.0001). Breaches of the virtual boundary were infrequent overall and occurred only within the initial adaptation phase. The majority of breaches were concentrated on Day 2 and Day 3, with 18 animals breaching the fence on each of these days, followed by a single animal on Day 4. A detailed daily distribution of collar-derived metrics for the herd (audio cues, electrical pulses) is provided in Table 2 and (SR) and CR) Table 3, which summarise the results of our statistical models. To provide a comprehensive picture of the data distribution, which was skewed due to many animals having zero interactions on certain days, we also report the overall median values. Across the entire study period, the median number of daily audio cues per animal was 0 (IQR: 0–2), and the median number of daily electrical pulses was 0 (IQR: 0–1).

According to the SR, the training protocol was effective in teaching the cattle to respect the virtual boundaries with the eShepherd collars. A linear mixed-effects model revealed a highly significant positive effect of the training day on the daily SR (F(11, 618,311) = 4771.46, *p* < 0.001), indicating that the herd’s performance significantly improved throughout the 12-day period, with the mean SR increasing from 0.50 on Day 1 to 0.83 by Day 12. In addition, the herd reached an average of 0.74 of SR on Day 10 of the trial.

The CR metric revealed complementary behavioural patterns. When analysed across all data the ratio showed a significant increase over the training period (Least Squares Means: Adaptation = 0.48 ± 0.014; Consolidation = 0.54 ± 0.015 and Validation = 0.63 ± 0.006; F-ratio 14,488.41, *p* < 0.0001).

A substantial proportion of the variation in learning performance was attributed to individual animal differences. The animal effect was highly significant (*p* < 0.001) and accounted for 39% of the total variance in the daily SR, highlighting the critical role of individual behaviour or inherent learning ability.

### 3.3. Age Determines the Learning Strategy and Trajectory

**Figure 3:**
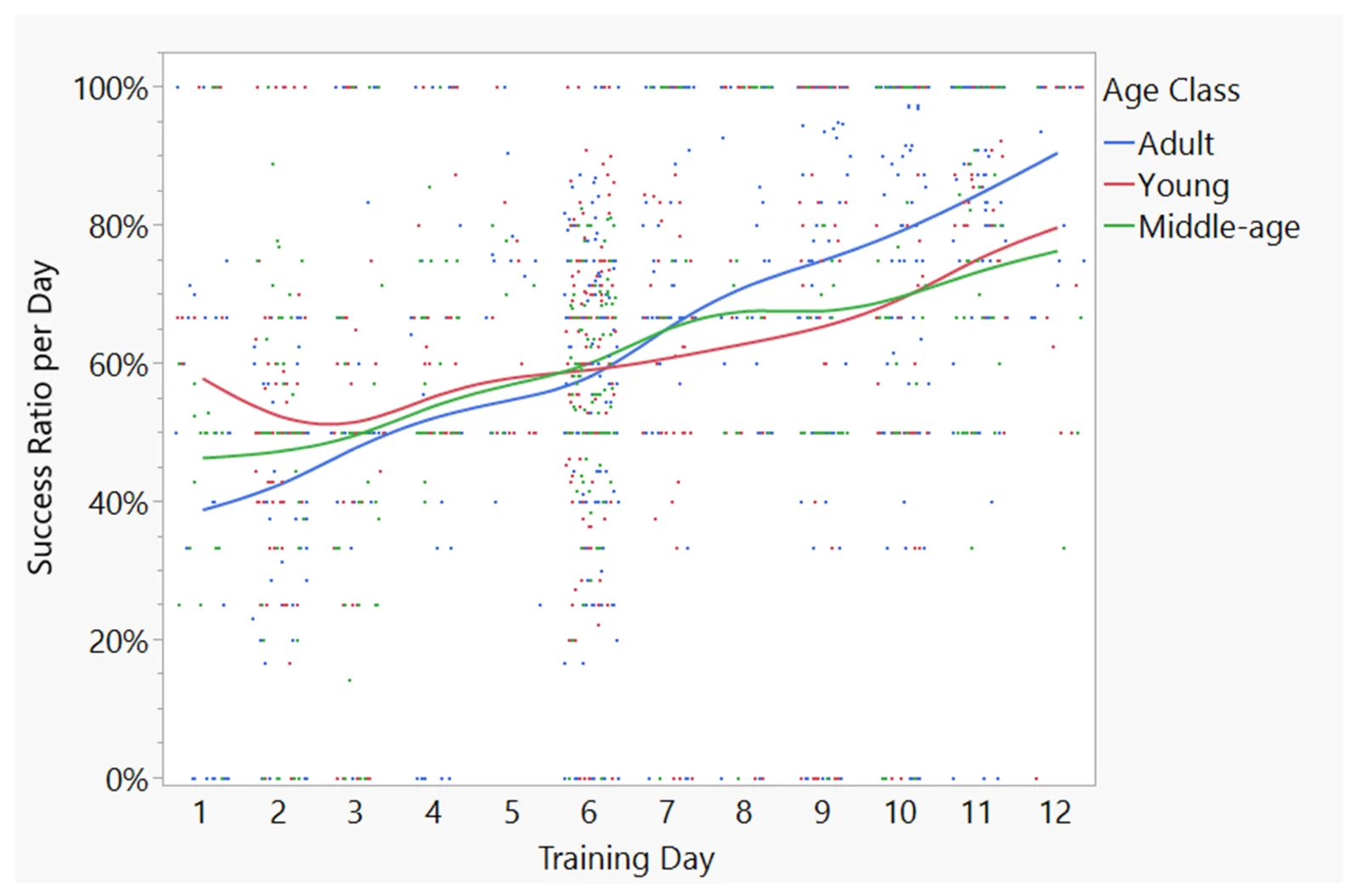
Success ratio per day (%) of the animals throughout the experimental period of 12 days with different age classes (Young: red, Middle age: green, and Adult: blue).

The learning trajectory to avoid the electrical pulse, measured by SR was significantly modulated by the age of the animals, as revealed by a significant interaction between 12 training days and age class (F(22, 1.7 × 10^6^) = 2741.51, *p* < 0.001; Figure 3).

Post hoc analysis using Tukey’s HSD of the learning trajectory showed distinct patterns. The young group of animals exhibited the most rapid initial adaptation, achieving a significantly higher SR (LSM ± SE; 0.8 ± 0.08) during the first training days compared to the other groups such as Middle age (0.5 ± 0.06) and Adult (0.45 ± 0.04). However, their performance showed greater variability and a less-consistent increase in later stages. The group of adults demonstrated a slower but steady learning curve. While their initial performance was lower than that of the young animals, they achieved the highest SR during the training’s final validation phase (0.9 ± 0.05) in comparison with young animals (0.7 ± 0.07). An intermediate learning pattern was notable in the middle age group, with decreases during specific stages including Day 6 in stage 4, but ultimately reaching a high level of performance by the end of the trial (0.7 ± 0.07). Analysis of the “cost to learn”, defined as the cumulative number of pulses for the animals (*n* = 102) that successfully reached 80% of SR criterion, revealed a non-significant difference across age classes (ANOVA: F (2, 99) = 2.67, *p* = 0.074). On average, the adult (mean ± SD, 7.9 ± 4.68) and young (7.9 ± 4.82) animals received numerically fewer cumulative pulses than the middle age (10.8 ± 3.92) animals.

### 3.4. Breed Influence on Learning Effect

**Figure 4:**
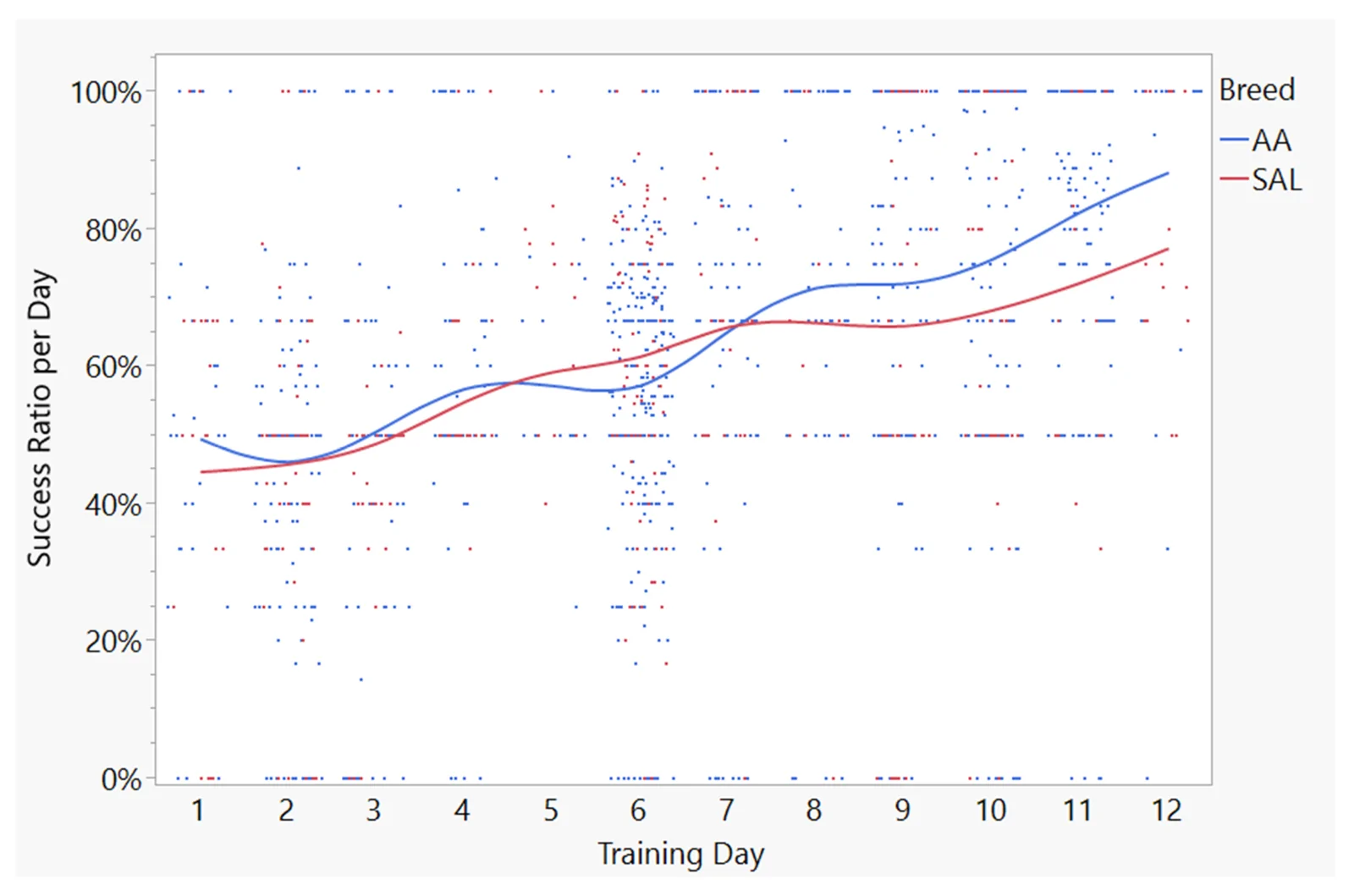
Success ratio (%) of the animals throughout the experimental period of 12 days interacting with breed (AA: Angus in blue; SAL: Salers in red).

Additionally, the linear mixed model showed that the breed had a significant effect on the learning process (Table 2 and Table 3). On average, Angus cattle received significantly fewer audio cues per day (mean ± SE; 0.90 ± 0.14) than Salers cattle (1.52 ± 0.23; *p* = 0.02). A similar pattern was observed for electrical pulses, with Angus receiving significantly fewer pulses (0.40 ± 0.03) than Salers (0.79 ± 0.05; (*p* < 0.0001). This behavioural difference was linked to learning performance, as revealed by a significant interaction between training day and breed for both SR and CR (*p* < 0.0001). While the Angus cattle showed a steeper curve for SR (Figure 4), overall data revealed a more complex dynamic. The Salers animals explored the inclusion zone more, despite a slower improvement in SR, and they exhibited periods of very high response accuracy during their frequent fence interactions. A post hoc analysis on Day 5 revealed that Salers had a significantly higher SR than Angus (*p* < 0.05), whereas on Day 12, this difference was inverted, with Angus performing better (*p* < 0.05) This indicates that the breed can influence the behavioural strategy and learning trajectory over the 12-day period.

### 3.5. Initial Aversive Stimuli and Subsequent Learning Rate

To test the hypothesis that a more intensive initial training experience would accelerate subsequent learning, we performed a simple linear regression. The analysis examined the relationship between the number of electrical pulses received during the initial adaptation phase (Days 1–2) and the individual learning rate during the consolidation and validation phases (Days 3–12). The regression revealed no significant correlation between these two variables (r^2^ = 0.001; *p* = 0.66). This finding suggests that the speed learning is more dependent on an animal’s intrinsic characteristic than on the intensity of early aversive conditioning.

## 4. Discussion

The primary finding of this study demonstrated that a mixed-age, mixed-breed herd of cattle successfully learned the concept of virtual fencing (VF) under grazing conditions. The system performance was robust and the GPS accuracy high, with a mean spatial deviation of 1.18 ± 0.47 m and 0.99 ± 0.29 m across movement and stationary tests, respectively. This is considerably more precise than the accuracies reported for other commercial VF systems observed by Versluijs et al. [25], which the authors described as a range from 3.3 to 2.5 m (stationary) and 10.8 to 6.8 m (mobile tests). Nevertheless, Grinnell et al. [26] also validated VF collars using a similar approach to the one used in the present study, resulting in an average deviation of 2.5 m.

### 4.1. General Learning and Habituation

We have been able to confirm our first hypotheses (H1 and H2); the herd demonstrated both behavioural habituation and successful performance-based learning. The performance-based learning was mirrored by a clear behavioural habituation observed by drone recording during the first five days of training. The most frequently observed behaviours were low-intensity reactions such as continuing grazing, slow turning, or head shaking (Scores 1 and 2). High-intensity reactions like jumping, bucking, or vocalising (Scores 3–5) were rare overall and became significantly less frequent after the third day of training. Our detailed examination using the ordinal logistic regression model confirmed this, showing that the most substantial change occurred between Day 3 and Day 4, with a highly significant decrease in the probability of observing higher-intensity reactions (χ^2^(5) = 13.51, *p* < 0.0001). This behavioural shift indicates a key learning and adaptation event after the initial 72 h. These findings align with a growing body of literature suggesting that, following a brief adaptation period, the VF technology does not cause long-term negative effects on cattle welfare [15,17,20]. In a recent meta-analysis, Wilms et al. [6] further supported this, concluding that VF is not associated with an impairment of welfare outcomes in cattle.

In our study the herd was evaluated every training day, and the collars data show that SR started to increase in Stage 3, specifically on Day 4 (mean ± SD, 0.60 ± 0.24). This value is lower than those values at a similar time point observed from Hamidi et al. [9], who reported a mean SR of 0.85. This difference can be explained by the smaller number of animals and size of the paddocks in their study (*n* = 12), which may have permitted more individual interactions with the VF and consequently a more rapid learning effect. In addition, the herd in their study maintained the SR constantly high until the end of the study, reaching 0.97. A further factor that may have contributed to the slower progress in learning in our trial is possibly the training schedule itself. The VF was only active during daylight hours and was deactivated at night, a protocol chosen to allow direct supervision. This intermittent exposure, in contrast to studies with 24-h activation, effectively halved the daily training time and may have slowed the consolidation of the learned association, thus requiring more calendar days to achieve a high level of performance.

### 4.2. Intrinsic Animal Factors in Learning Trajectories

Our study utilised a multi-layered analysis, combining high-frequency collar data with drone behavioural observations, providing a comprehensive picture of the adaptation process. The congruence between these two data sources, observing that the number of adverse reactions decreased as the SR began to increase, highlights the importance of integrating evaluation methods. We observed that the number of animals crossing the boundary decreased as the animals became habituated to the system in the first four days, which corresponded with the period when the SR started to increase. The complexity of the learning process with VF involves cognitive and behavioural components. It is beneficial to compare observational data with collar parameters in order to quantify holistic learning at least during the first days of interaction with VF technology [9]. This integrated approach allowed us to move beyond general learning trends and investigate the intrinsic animal factors that drive individual differences (H3). Our results revealed that intrinsic characteristics, particularly age, dictate learning strategies rather than a simple difference in learning ability. Young animals showed a rapid learning rate with a high SR during the first days of interaction with virtual fencing in comparison to the adults; however, the latter demonstrated a more consistent learning curve during the experimental days, which was not observed with the young in the same period. This difference in learning strategy was further illuminated by the “cost to learn” analysis. Among the animals that successfully learned, age did not have a significant statistical effect on learning efficiency. Although the learning trajectories of young and adult animals were divergent, both groups required a similar number of cumulative pulses to reach the 80% SR criterion (approximately eight pulses each). The middle-aged group received numerically more cumulative pulses (11 pulses), but this difference was not significant. This suggests that different behavioural pathways, such as the rapid initial learning of the young animals versus the slower, more consistent curve of the adults can ultimately lead to a comparable level of learning efficiency. Additionally, we demonstrated that age groups differed in SR and number of electrical pulses throughout the trial. This contrasts with the findings of Confessore et al. [18], who investigated the learning ability of cattle with virtual fences and compared between younger and adult cows, and no difference was observed between age groups. Moreover, the SR reported in their study tended to increase after two days of training, demonstrating an initial adaptation of the animals to the technology. Campbell et al. [5] did not find differences in the learning process between the age groups, but they observed that young animals demonstrated an increase of activity behaviour per day throughout the trial, which corroborates our findings regarding the young animals exploring the area of VF faster with higher initial SR in comparison with the adults. A study from Verdon and Rawnsley [7] that examined the age’s influence on the ability to learn and retain the association between stimuli from the VF system observed, contrary to their initial hypothesis, that younger animals with higher neural plasticity did not necessarily learn faster. For instance, older heifers often show faster and more consistent learning during training compared to younger, more active animals [7,13,18]. One explanation for this performance of the adult animals may be explained by the reproductive status as late pregnancy and maternal hypervigilance can allow these animals to maximise attention to and protection against potential dangers, and consequently learn more quickly to control the stimuli through behavioural changes. In addition, older animals have spent more time exposed to the environments of pasture-based systems, such as moving to new areas and having contact with conventional electric fences. Such exposure can strengthen cognitive skills by inducing higher levels of neuronal plasticity, enabling the animal to cope better with new environmental challenges like VF [7,10].

Beyond the clear influence of age, our results also revealed differences in the learning effect caused by other intrinsic factors including breeds, individual behaviour, and social dynamics. The adaptation of cattle to VF systems is a complex process influenced by breed characteristics, individual variation, and social dynamics. In accordance with our findings, Keshavarzi et al. [10] noted that the breed can influence the behavioural strategy and learning trajectory. The collar data demonstrated that Salers explored the inclusion zone more than the Angus breed, and they exhibited periods of very high response accuracy during their frequent fence interactions. This suggests that the Angus breed adopted a more consistent and cautious learning strategy, and the Salers engaged in a more active learning style based on trial and error. A significant interaction was observed between training day and breed, indicating that the two breeds followed different learning trajectories. Angus cattle, for instance, exhibited a steeper increase in their SR during the final days of trial, suggesting a more efficient consolidation of learning. However, we interpreted this finding with caution, as the differences might be more related to individual ability to adapt to VF technology and not directly to the breed in this case, due to two principles: first the unequal number of individuals in each group, Angus (*n* = 125) and Salers (*n* = 46), which also showed high variance in SR (mean ± SD; 0.65 ± 0.29, and 0.61 ± 0.29, respectively). Second, both breeds are considered to have a similar individual behaviour and were managed as a single, cohesive herd, suggesting that other factors may be more influential. Different studies demonstrated the ability to learn the association between audio cues and electrical stimuli in a variety of breeds including Angus, Holstein–Friesian, Fleckvieh, and Rarámuri Criollo [6,13,19]. Significant individual variation exists within those groups, with factors such as age playing a major role as mentioned also in our present study [7,13,18,19].

Our study further showed that the individual behaviour had a significant effect on the animals’ learning process, with an average of 22.32 ± 1.05 (mean ± SE) and 9.03 ± 0.32 audio cues and electrical pulses, respectively, throughout 12 days in total. We observed that one animal accumulated a maximum of 101 audio cues and 11 pulses, while four animals had no interaction with VF at all. We assume those animals that interacted with the VF receiving many audio cues but only a few pulses, were affected in their learning process by the herd behaviour, where the animal learns the system observing the behaviour of others interacting with the VF. This is underlined with the GPS accuracy test of the present system; it could be assumed that some animals receive earlier audio cues even if they are near together. We were unable to study the behaviour of the cattle in detail during the training period; we instead focused on five different behaviours with drone observations in the first five days and we were able to observe more active animals in the initial phase of the study exploring the area near the virtual boundary attracted by fresh pasture beyond the VF. In addition, some animals were more active in exploring the virtual boundaries than others. In a large herd such as in our study, we could demonstrate a significant individual animal effect on the SR and CR. Some studies described that the trained animals could express individual variation in learning outcomes [23,24]. In herd dynamics, leadership is more a matter of personality than of simple dominance, and the animals that take the lead are typically not the most social, but rather the most bold and explorative individuals [19].

The presence of the herd mates enhances the ability to become familiar with the virtual fencing system through social learning [23,27]. Social learning, such as observing the avoidance reactions of herd mates, can allow an individual to learn the location of the virtual boundary while minimising its own exposure to the aversive electrical pulse [10]. Nevertheless, breed and within-breed individual differences in social- and asocial-learning mechanisms could potentially influence an individual’s adaptation to the technology [24,28].

### 4.3. Mechanisms and Metrics of Learning

To investigate a potential learning mechanism, we tested the hypothesis (H4) that a higher intensity of initial aversive stimuli would accelerate subsequent learning. However, our analysis found no significant correlation between the number of pulses received in the adaptation phase and the subsequent individual learning rate. This finding suggests that the speed of learning is an intrinsic characteristic of the animal rather than a direct consequence of the intensity of early aversive conditioning.

Relying solely on the SR can be misleading, as a high SR may not distinguish between an animal that has learned the audio-cue association and one that has simply adopted a strategy of spatial avoidance of VF [6,9,11]. The findings of these authors support that the SR solely is not sufficient to evaluate the learning progress with VF, and that the trend and magnitude of the CR can indicate the confidence of the animals to interact with the technology.

Our results also demonstrated a CR that was consistently lower than that reported in studies with smaller groups [4,9], an effect we attribute to the social dynamics of a large herd. In a large group, individual interactions with the VF can be heavily influenced by “herd mentality”, where many animals learn to avoid the virtual boundary simply by observing the negative reactions of their herd mates. This social learning effect decreases the overall number of interactions. We assume that while the animals in our study understood the VF system, their confidence to interact was limited due the amount of the individuals and these social dynamics, resulting in a lower overall CR. This indicates that when animals did choose to approach the boundary, their ability to respond correctly to the audio cue (i.e., their response accuracy) improved significantly with experience. The increase of CR is typically interpreted as animals gaining confidence in their ability to control the acoustic signals, and our findings suggest that the CR is also highly sensitive to social context [9]. These contrasting results suggest that successful training comprises two behavioural components: a strategic decision to avoid the fence zone entirely, and an improved tactical response when an interaction does occur. The empirical criteria used in the CR formula, such as the value of 20 interactions, may therefore not be directly comparable across studies with vastly different herd sizes, individual variations, and social structures.

This finding has direct implications for training protocols, suggesting that an increased duration of the training period in a large herd may be necessary. Previous studies conducted experiments involving a three-day training period for cattle to adapt to VF systems [22,29]. These authors found that the heifers had successfully interacted with the virtual boundary, that most associative learning occurred within the first 24 h of exposure, and that the animals required an average of only three audio events to form a reliable association between the sound and the electrical stimulus.

A brief event occurred on Stage 4 of the trial period, providing a unique insight into the conflict between learned avoidance and strong motivational drivers due to an accidental placement of a feeding site near the VF boundary. The number of animals interacting with VF consequently increased, and the mean daily SR dropped (0.56 ± 0.21), which demonstrated a clear, albeit temporary, disruption of the learned behaviour. A similar effect was observed by Verdon et al. [29], who described social facilitation of animal responses to the VF in an adverse event, where the animals followed a leader into the exclusion zone. These results showed that the cattle present a herd behaviour in occasions of motivational challenges, where not only a single animal acts but the collective. In addition, the provision of feed during the training period requires careful consideration of time and place to not force the animals to act as herd close to the VF area, but to instead learn by the exploratory method.

### 4.4. Limitations and Implications

Several considerations of the present study should be included in further trials. First, the research was conducted at a single site with a specific herd composition, which may limit the generalisability of the findings. Second, the behavioural analysis, while insightful, did not allow the animals to be identified individually, restricting the analysis to group-level trends. Third, our training protocol was limited to daylight hours, with the VF system being deactivated at night. While this allowed for essential supervision, this intermittent exposure differs from a continuous, 24-h commercial application and likely extended the number of days required for the herd to reach proficiency. Additionally, the 12-day training period may still be insufficient for some individuals in a large herd to gain full confidence with the VF system, presenting a potential management challenge.

An effective VF training protocol must account for these diverse internal and external factors to ensure that every animal in the herd establishes a predictable and controllable interaction with the technology [10,19,27,28]. This social facilitation can improve the herd’s overall adaptation rate and minimise individual shocks, but it can also pose challenges, such as socially motivated breaches where the desire to maintain herd cohesion or to reach resources overrides the learned avoidance of the virtual boundary.

Finally, while our tests confirmed a high overall GPS accuracy, this inherent inaccuracy can still pose an interpretive challenge, particularly in a large, dense herd. It is possible that the system occasionally delivered an audio cue to an animal based on a slightly inaccurate GPS position, especially when multiple animals were clustered near the boundary. Therefore, some of the observed individual variation in the number of audio cues or in the SR could be partly attributable to random GPS error rather than purely to animal behaviour. This potential confound between true individual behavioural differences and system-level spatial inaccuracy is a known challenge in VF research and warrants further technological refinement.

## 5. Conclusions

This study demonstrates that cattle adaptation to virtual fencing is a complex process, fundamentally shaped by intrinsic animal characteristics, particularly age, breed, and individual behaviour. Our results reveal distinct learning strategies, with younger animals acting as rapid but inconsistent learners and adults as slower but more reliable finishers. The Salers cattle demonstrated more explorative behaviour through more frequent interactions with the VF, while the Angus breed adopted a more consistent and cautious learning strategy. The analysis of high-frequency collar data and behavioural observations provided a comprehensive picture of this adaptation process, highlighting the importance of social learning dynamics within a large group. Understanding these different individual and social learning pathways is essential for developing more effective, efficient, and welfare-centric training protocols for virtual fencing technology.

## Figures and Tables

**Table 1:** Cattle behaviour from drone footage during contact with virtual fences following a collar cue (referring to an acoustic signal or an electrical pulse) described as reaction score and the definition adapted from Hamidi et al. [9].

Reaction Score	Definition
1	Animal continues to graze or walk slowly (<3 steps) while turning around; away from the VF line; causing the signal to stop
2	Head shaking, walking (>3 steps), jumping (only with front legs); away from the VF line; causing the signal to stop
3	Running (trot or canter); jumping; bucking away from the VF line, causing the signal to stop
4	Running (trot or canter); mooing with tongue out; trying to take off the collar, bucking away from the VF line, causing the signal stop
5	Breaking through the VF line

**Table 2:** Linear Mixed Models (LMMs) for raw interaction metrics of collar parameters, with overall test statistics for each main effect and the Least Squares Means (LSM) ± Standard Error (SE) for each level.

AC	F-Value (DF, DFden)	*p*-Value	EP	F-Value (DF, DFden)	*p*-Value
Breed	F(1, 169.1) = 5.52	0.02	Breed	F(1, 171.1) = 40.25	<0.0001
	LSM ± SE			LSM ± SE	
AA	0.90 ± 0.14		AA	0.40 ± 0.03	
SAL	1.52 ± 0.23		SAL	0.79 ± 0.05	
Age Class	F(2, 169.1) = 9.51	<0.0001	Age Class	F(2, 171.1) = 58.46	<0.0001
Young	0.49 ± 0.19		Young	0.70 ± 0.04	
Middle-aged	1.77 ± 0.26		Middle-aged	0.18 ± 0.04	
Adult	1.39 ± 0.19		Adult	0.92 ± 0.06	
Training Day	F(11, 1.7 × 10^6^) = 9541.61	<0.0001	Training Day	F(11, 1.7 × 10^6^) = 26,080.61	<0.0001
1	1.22 ± 0.14		1	0.60 ± 0.03	
2	3.79 ± 0.14		2	1.81 ± 0.03	
3	2.01 ± 0.14		3	1.10 ± 0.03	
4	0.91 ± 0.14		4	0.37 ± 0.03	
5	6.81 ± 0.15		5	2.05 ± 0.04	
6	4.96 ± 0.14		6	2.90 ± 0.03	
7	1.56 ± 0.14		7	0.45 ± 0.03	
8	0.68 ± 0.14		8	0.17 ± 0.03	
9	1.58 ± 0.14		9	0.49 ± 0.03	
10	1.96 ± 0.14		10	0.58 ± 0.03	
11	1.83 ± 0.14		11	0.43 ± 0.03	
12	0.54 ± 0.14		12	0.13 ± 0.03	

AC: Audio cue; EP: Electrical pulse; AA: Angus; SAL: Salers.

**Table 3:** Linear Mixed Models (LMMs) for calculated learning-performances ratios of collar parameters, with overall test statistics for each main effect and the Least Squares Means (LSMs) ± Standard Error (SE) for each level.

SR	F-Value (DF, DFden)	*p*-Value	CR	F-Value (DF, DFden)	*p*-Value
Breed	F(1, 166.4) = 10.47	0.0015	Breed	F(1, 168.0) = 7.16	0.0082
	LSM ± SE			LSM ± SE	
AA	0.69 ± 0.02		AA	0.11 ± 0.01	
SAL	0.59 ± 0.02		SAL	0.07 ± 0.01	
Age Class	F(2, 168.4) = 91.02	<0.0001	Age Class	F(2, 170.7) = 1.39	0.2509
Young	0.91 ± 0.02		Young	0.09 ± 0.01	
Middle-aged	0.48 ± 0.03		Middle-aged	0.07 ± 0.01	
Adult	0.52 ± 0.03		Adult	0.10 ± 0.01	
Training Day	F(11, 618,311) = 4771.46	<0.0001	Training Day	F(11, 618,334) = 1851.75	<0.0001
1	0.64 ± 0.02		1	0.09 ± 0.01	
2	0.46 ± 0.02		2	0.09 ± 0.01	
3	0.50 ± 0.02		3	0.05 ± 0.01	
4	0.60 ± 0.02		4	0.08 ± 0.01	
5	0.53 ± 0.02		5	0.26 ± 0.01	
6	0.37 ± 0.02		6	0.06 ± 0.01	
7	0.67 ± 0.02		7	0.12 ± 0.01	
8	0.64 ± 0.02		8	0.06 ± 0.01	
9	0.61 ± 0.02		9	0.08 ± 0.01	
10	0.62 ± 0.02		10	0.10 ± 0.01	
11	0.69 ± 0.02		11	0.12 ± 0.01	
12	0.74 ± 0.02		12	0.07 ± 0.01	

SR: Success ratio; CR: confidence ratio; AA: Angus; SAL: Salers.

## Data Availability

Dataset available on request from the authors.

## Abbreviations

The following abbreviations are used in this manuscript:

AA	Angus
AC	Audio cue
BCS	Body condition score
CR	Confidence ratio
EP	Electrical pulse
SD	Standard deviation
SE	Standard error
SAL	Salers
SR	Success ratio
VF	Virtual fencing
